# Behind closed doors: a qualitative study exploring the content of fertility discussions between oncologists and their adolescent and young adult cancer patients from the perspective of oncologists at an NCI-designated comprehensive cancer center

**DOI:** 10.1007/s00520-025-09269-0

**Published:** 2025-03-21

**Authors:** Julia Stal, Charleen I. Roche, Serena Y. Yi, David R. Freyer, Rachel C. Ceasar, Sue E. Kim, Joel E. Milam, Kimberly A. Miller

**Affiliations:** 1 Department of Population and Public Health Sciences, Keck School of Medicine, University of Southern California, 1845 N Soto St. Third Floor, Los Angeles, CA 90033 USA; 2 Cancer and Blood Disease Institute, Children’S Hospital los Angeles, Los Angeles, CA USA; 3 Departments of Pediatrics and Medicine, Keck School of Medicine, University of Southern California, Los Angeles, CA USA; 4 USC Norris Comprehensive Cancer Center, Los Angeles, CA USA; 5 Department of Epidemiology and Biostatistics, Department of Medicine, Chao Family Comprehensive Cancer Center, University of California, Irvine, CA USA; 6 Department of Dermatology, Keck School of Medicine, University of Southern California, Los Angeles, CA USA

**Keywords:** Oncofertility, Cancer care delivery, Adolescents and young adults, Reproductive health, Cancer survivorship

## Abstract

**Purpose:**

To qualitatively explore the content of fertility discussions between oncologists and their adolescent and young adult (AYA; 15–39 years) cancer patients from the perspective of oncologists at an NCI-designated comprehensive cancer center.

**Methods:**

We recruited oncologists of various specialties employed at an NCI-designated comprehensive cancer center in California who treat AYAs at risk for infertility. We collected demographics and fertility-related information (if they discuss fertility with AYA patients and level of confidence doing so) via REDCap prior to conducting a semi-structured interview via HIPAA-compliant Zoom. Audio files were transcribed verbatim and reviewed for themes using an inductive codebook thematic analysis approach.

**Results:**

Oncologists (*n* = 12) were female (66.7%), of White or Asian race (41.7% each), and were on average in practice for 14.3 years (SD = 6.7). All endorsed discussing fertility with AYAs and were on average somewhat/fairly confident doing so. The detail with which oncologists reported discussing fertility with AYAs varied substantially and only some reported discussing costs associated with fertility preservation. Oncologists also reported assorted information they always mention, mention on a case-by-case basis, avoid, or feel is not necessary when discussing fertility.

**Conclusion:**

This study provides a detailed description of information delivered by oncologists during fertility discussions to their AYA patients, revealing unstandardized oncofertility counseling. Fertility discussions were described to vary widely in depth and content, suggesting adherence to clinical practice guidelines limited. Interventions to increase provision of guideline-concordant counseling are needed to provide actionable pathways by which AYAs can proactively mitigate adverse reproductive health outcomes.

**Supplementary Information:**

The online version contains supplementary material available at 10.1007/s00520-025-09269-0.

## Background

In 2023, there will be an estimated 85,980 new cancer cases diagnosed among adolescents and young adults (AYAs; 15–39 years) in the United States [1]. Treatment modalities such as chemotherapy and radiation therapy, while effective, are known to cause iatrogenic infertility among AYAs [2–4]. Fortunately, this impairment can be mitigated with timely fertility discussion and preservation among both males (e.g., sperm banking) and females (e.g., egg freezing) to ensure that cancer does not have lasting implications on their reproductive futures [5–7]. Organizations such as the American Society of Clinical Oncology (ASCO), the American College of Obstetricians and Gynecologists (ACOG), and the American Society for Reproductive Medicine (ASRM) have developed clinical practice guidelines, committee opinions, and/or descriptions of medical and ethical considerations with regard to fertility and cancer to serve as a framework and facilitate comprehensive fertility-related care delivery [2, 5–11].

ASCO clinical practice guidelines indicate that fertility discussions should occur upon confirmation of diagnosis and include infertility risk, referral to a reproductive specialist, preservation options, costs, and pregnancy risks as necessary for the patient [5–7]. Timely discussions are particularly relevant for AYAs who are in the prime of their reproductive years and report fertility-related distress following their cancer treatment [12]. Despite guidelines, fertility discussion rates among AYAs are suboptimal and a lack of fertility information is a prominent unmet need for this population [12–18]. Over 80% of young cancer patients endorse a desire to have children in the future; over three-quarters report clinically significant distress after treatment due to fertility impairment and have clinically significant decisional conflict scores; and nearly half report moderate to high reproductive concerns [14–16, 19, 20]. However, adequate fertility counseling remains elusive in this population [12–18].

Oncofertility discussions between cancer patients and their providers are poorly understood. Nearly one-third of survivors report dissatisfaction with either the amount or quality of information received regarding the effects of treatment on their fertility [21]. Furthermore, AYAs who report receiving a fertility discussion also report greater reproductive concerns, suggesting counseling may overlook components that can better guide patients’ fertility decisions [14]. Existing research predominantly characterizes fertility discussions through a dichotomous lens of whether they occurred, as reported either through medical records or patient self-report [22, 23]. However, such methodology does not identify explicit areas of fertility counseling that require intervention because it broadly characterizes the frequency of fertility discussions while failing to capture oncofertility care delivery practices, information relayed, and provider involvement in oncofertility care. Thus, we lack a granular description of fertility discussions, limiting our understanding of information provided and guideline-concordance.

The purpose of this study is to qualitatively explore the content of fertility discussions between oncologists and their AYA cancer patients from the perspective of oncologists at an NCI-designated comprehensive cancer center. We will comprehensively describe oncofertility counseling at an NCI-designated comprehensive cancer center to identify patterns and subsequent gaps in care delivery. Understanding the nuances surrounding oncofertility counseling can inform the targeted development of interventions to improve patient-provider communication, detail gaps in medical knowledge to inform provider training, and ensure patients are equipped with the knowledge needed to engage in informed decision-making surrounding their reproductive futures.

## Methods

### Participants and recruitment

We recruited oncologists of various specialties who treat AYAs with cancer types likely to result in treatment-related infertility and who were employed at an NCI-designated comprehensive cancer center in Southern California. This institution does not have a central oncofertility patient navigator and disease teams are expected to address fertility on their own. However, this institution has an AYA program which supports patients and providers in receipt and delivery of supportive care that targets the unique needs of AYAs. Patients can contact the AYA program on their own to make an appointment, or can be referred by a provider, though no referral is needed. Convenience sampling was used to identify eligible oncologists through clinical co-investigators involved in the study [24–26]. Opportunistic sampling was also used if opportunities emerged throughout data collection (i.e., if a participant recommended another oncologist who engages in cancer care with AYAs at risk for infertility) [24].

Oncologists were contacted by the Principal Investigator (PI; JS) via email for participation in the study. An information sheet with the elements of informed consent was provided to interested oncologists, who then provided verbal agreement to participation and recording before beginning the interview. Upon interview completion, oncologists received a $25 gift card and a resource sheet including information on costs associated with fertility preservation, fertility providers for patient referral at their place of practice, and external patient resources for fertility preservation funding and advocacy. Recruitment was open for 8 months, from April to November 2023, until data saturation was reached. A follow-up email was sent to oncologists who were contacted for participation but did not initially respond. In total, 27 oncologists were contacted and 12 participated, resulting in a recruitment rate of 44.4%. This study was conducted according to the guidelines of the Declaration of Helsinki and was approved by the University of Southern California (USC) Institutional Review Board (IRB; UP-22–00847).

### Data collection

Using a brief online HIPAA-compliant REDCap survey prior to the interview, oncologists were asked to report their demographic information (i.e., sex, race/ethnicity, specialty, years of experience) and fertility-related information (i.e., if they discuss fertility with their AYA patients [*yes, no, not sure*] and how confident they feel doing so using a 4-point Likert scale ranging from 1 “slightly” to 4 “completely”). All study procedures were conducted in English. Qualitative interviews were led by the PI (JS) with a co-interviewer present (CIR or SYY) and were conducted and audio recorded via HIPAA-compliant Zoom. Audio files were sent to an external service for verbatim transcription and were deleted immediately after transcription. Interviews and de-identified transcripts were stored on a HIPAA-compliant secure server. Interviews with oncologists were conducted using questions and probes from a semi-structured interview script designed to elicit oncologists’ subjective experiences discussing fertility with their AYA patients (Appendix). The estimated time for completion of the demographic survey was under 5 min and under 60 min for the qualitative interview.

### Analytic plan

#### Demographics

Frequencies were analyzed to describe oncologists who participated in the study, to determine whether they discuss fertility with their AYA patients, and to measure their level of confidence doing so.

#### Qualitative

Data/thematic saturation was achieved through 12 interviews, given the homogenous nature of our participant population [25, 26]. Achievement of data saturation through the recruitment of 12 participants is evidence-based and has been deemed sufficient for capturing perceptions among a group of homogenous individuals [25, 26]. We (JS, CIR, SYY) reviewed transcripts for patterns and conducted an inductive codebook thematic analysis following the Braun and Clarke (2006) six phase method for establishing trustworthiness during thematic analysis [27]. First, the team (JS, CIR, SYY) took theoretical/observational notes (i.e., memoing) on initial interviews from which an initial set of codes was generated. Then, we applied initial codes to new interview transcripts. Data and memos were reviewed and coded for themes. Codes were developed through an iterative and reflective process between the team. We assessed the frequency of themes during interviews and included themes mentioned in the interviews that were not specified in the original interview guide. The team reviewed codes and themes as they were developed, considered alternatives, and revised before arriving at a set of final thematic codes. The codebook was subsequently refined and adapted as needed. We then uploaded and analyzed transcripts using ATLAS.ti (Version 22.0.1), a computer-assisted qualitative analysis software program, to facilitate the consistent application of themes.

## Results

The sample consisted of 12 oncologists who were primarily female (66.7%), of White or Asian race (41.7% each), and were on average in practice for 14.3 years (SD = 6.7, range 8–29 years; Table 1). Our response rate of 44.4% is consistent with, or greater than, prior research among clinician samples [28–30].

**Table 1:** Participant characteristics

	***N* (%) or M (SD)**
Sex	
Female	8 (66.7)
Male	4 (33.3)
Race/ethnicity	
White	5 (41.7)
Asian	5 (41.7)
Hispanic	1 (8.3)
Other	1 (8.3)
Years in practice	
Mean	14.3 (6.7)
Range	8–29
Discuss fertility	12 (100.0)
Confidence discussing fertility	2.9 (0.9)
Slightly (1)	1 (8.3)
Somewhat (2)	2 (16.7)
Fairly (3)	6 (50.0)
Completely (4)	3 (25.0)
**Interview**	**Specialty**
1	Medical Oncology, Breast Oncology
2	Medical Oncology, Genitourinary Oncology
3	Gynecologic Oncology
4	Medical Oncology, Sarcoma Oncology
5	Medical Oncology, Genitourinary Oncology
6	Medical Oncology, Sarcoma and Melanoma Surgery
7	Urologic Oncology, Urology
8	Medical Oncology, Solid Tumor and Cutaneous Oncology
9	Neuro-Oncology
10	Medical Oncology, Sarcoma and Melanoma Surgery
11	Gynecologic Oncology
12	Medical Oncology, Gastrointestinal Oncology

### Patient population seen

Some oncologists reported that AYAs comprise less than 15% of their practice, while others reported that AYAs comprise 20–40% of their practice. Oncologists reported treating a range of cancer types. In aggregate, these included genitourinary and urologic (e.g., bladder, prostate, renal, penile, kidney, testicular, urethral, scrotal), gastrointestinal (e.g., colon, rectal, biliary, esophageal, gastric, neuroendocrine, pancreatic, liver, bile duct), gynecologic (e.g., ovarian, uterine, endometrial, cervical, vulvar, vaginal), skin, brain, lung, and breast malignancies and retroperitoneal sarcomas. All oncologists reported that they discuss fertility with their AYA patients and were on average “somewhat” to “fairly” confident discussing fertility (mean = 2.9, SD = 0.9, range 1 to 4 where 4 is most confident), with 50% of oncologists reporting that they are “fairly confident” (confidence level is described in Table 1).

**While all oncologists reported discussing possible infertility with their AYA patients, the depth of these discussions varied substantially **(Table 2). All oncologists reported informing AYAs of possible iatrogenic infertility, many discussed options to preserve fertility, some discussed fertility sparing options and the need for timely fertility care, some referred patients to fertility specialists, few referred patients to social work, and fewer discussed risks and benefits with patients or referred patients to the AYA program. Regarding fertility-sparing options, one gynecologic oncologist stated:

Say for cervical cancer, early-stage cervical cancer, the standard recommendation is hysterectomy, but there’s ways to remove just the cervix and leave the uterus in place. So, there are things that can be done for patients that desire fertility, and the same thing for endometrial cancer. The standard of care is hysterectomy but that cancer can also be treated with an IUD…there are variations and there are things we do differently depending on patients’ fertility goals. (Gynecologic oncologist)

**Table 2:** Infertility and fertility preservation counseling*

Summary of information	Quote	Oncologist
Impact to fertility	“What is the duration of that therapy and then how would that affect their ability to have children when we’re finished.”	Medical oncologist
Referral to fertility specialist	“Sometimes we’ll ask the patients, are you still planning to have children, are you still interested in having children? Have you considered this? Is there someone you need to talk to, a partner or anyone about this? And then depending on their response we would say, okay, well, if this is something you are still interested in, we want you to go meet with the REI doctors, or the OB to discuss what the options are.”	
Fertility preservation options, speed	“If we’re going to work with fertility specialists, we would want that to happen as soon as possible. If we’re going to do some sort of egg harvest or any kind of intervention for preserving their future child-bearing capabilities, we would want that to happen quickly.”	
Impact to fertility	“Once you start talking about treatment and risks and adverse events, you do mention fertility.”	Medical oncologist
Fertility preservation options, referral to social work	“Then if it’s something they’re interested in, usually you’ll say have you thought about sperm banking? And refer them to social work.”	
Impact to fertility	“If it’s something that’s going to involve the removal of at least one of their ovaries, then once we start talking about that we’re talking about if it’s possible to do fertility sparing procedures and what that would entail.”	Gynecologic oncologist
Fertility-sparing options	“If it is something that is avoidable, we talk about what their options are and talk about what option they can have to avoid losing their fertility. Say for cervical cancer, early-stage cervical cancer, the standard recommendation is hysterectomy, but there’s ways to remove just the cervix and leave the uterus in place. So, there are things that can be done for patients that desire fertility, and the same thing for endometrial cancer. The standard of care is hysterectomy but that cancer can also be treated with an IUD. Most of our patients who desire future fertility we put an IUD in and then when they’re done with childbearing, we take it out. So, there are variations and there are things we do differently depending on patients’ fertility goals.”	
Impact to fertility	“I usually just say that one of the side effects of your systemic therapy is that there is a risk of infertility. All therapy is typically geared towards rapidly dividing cells and sperm or ovaries are rapidly dividing cells. The women that are closer to menopause can, so if you’re in the upper range of AYAs there’s a higher risk for you to develop menopause and therefore infertility, which is the most common cause, and for males the most common is just killing off of the sperm or becoming azoospermic, and those risks are X percent…At the end of that discussion I’ll typically say a lot of the literature is very gray. It’s not really clear what the true percentage is. I would say that the fact that you’re on this therapy which has a high percentage of infertility, I can tell you that some patients actually have had children after getting this therapy. However, it’s important for you to take precautions against that.”	Medical oncologist
Fertility preservation options, speed	“If you can get that fertility preservation, either harvesting your eggs, I would probably wait about 2 weeks, but if you get in very quickly we might be able to harvest your eggs. Or if you’re a male, we can get this on board right away and get you with a sperm bank and get everything approved very quickly.”	
Impact to fertility	“In terms of their fertility care, I will discuss the chances of their decreased fertility based on which chemotherapy regimen we’ve planned.” “I talk to them about their chances, how their treatment would decrease their chances, and then tell them about fertility preservation and what that entails.”	Medical oncologist
Fertility preservation options, referral to social work	“I will ask them if they are interested in fertility preservation, which for me, because I see mainly male patients, is sperm banking, and if they’re interested, we will generally have our social workers meet with them and help them in terms of resources that we give them.”	
Impact to fertility, referral to fertility specialist, risk/benefit ratio	“So firstly outline the stage of the cancer, the treatment plan, and then you would explain to the patient that there’s a risk of infertility and the implications of that, the type of symptoms to anticipate, and essentially refer them to a specialist to discuss options for preservation and then explain the risks and benefits of either delaying treatment for that purpose versus not.”	Medical oncologist
Impact to fertility, fertility preservation options, referral to sperm bank	“So typical will be testicular patients who are undergoing a radical orchiectomy and there’s a potential for decrease in potential or there’s a new diagnosis of infertility or subfertility within that patient. So we send them for a semen analysis and also semen banking. I will give them resources to bank sperm prior to the orchiectomy so that they can maximize their counts and quality of their sperm, and then the others are chemotherapy, so those with metastatic testicular cancer are referred to my medical oncology colleagues and the same discussion except that’s a little bit usually quicker…So those are the testicular cancer patients. The others are rare, where someone young has bladder cancer and we’re having to do a radical cystoprostatectomy, removing all their prostate and seminal vesicles, so it’ll be difficult for them to have children afterwards. So again, we’ll send them for a sperm banking prior. So those are young patients with bladder cancer undergoing radical cystoprostatectomy. Then also some patients with prostate cancer, we’ll just mention it. It is extremely rare if someone in an age range for prostate cancer wants to preserve their fertility because they’re both prostatectomy and cystoprostatectomy, we’re removing the bladder and/or prostate, they will no longer be able to have a normal sort of fertility. They’re essentially undergoing vasectomies.”	Medical oncologist
Speed	“We try to get them in as soon as possible and initiate that conversation from the initial consultation, but try to get them in to bank sperm as soon as possible so that we can start chemo prior.”	
Impact to fertility, fertility preservation options	“The discussion is part of the cancer history, we’re taking a complete history and physical. Then you get to the social history, do you have children? And if they say yes, how many? Do you desire any more children? You’re going to need an orchiectomy, and then I get into the possibilities of infertility…I highly encourage it prior to the orchiectomy and certainly prior to chemotherapy, as the chemotherapy will 100% get rid of all the sperm. So if you want to preserve that, you’d want to have sperm banking done prior to chemo.”	
Impact to fertility, risk/benefit ratio	“We have a conversation about our treatment goals first. If you’ve been diagnosed with a dangerous form of cancer, my job as your oncologist is to try to treat you with the best options that we have available in controlling that disease as well as possible. For the patients with earlier stages of disease, our goal may be to cure you, in which case we may be aiming very high, and for that reason, we may be willing to take more important risks. On the other hand, if we think there’s a very low chance of cure, does that change what kind of risks and what kinds of things we’re willing to do? This is kind of generally how I approach any patient in terms of discussing treatments. Looking at those treatment options, we’ll then discuss the risk/benefit ratio, what are the potential side effects associated with certain regimens versus another, and how likely do I think those are going to happen, and I will fully explain in great detail as much as possible what they likely may expect and also what could happen in the most rare worst case scenario, because I want them to be fully informed of that.”	Medical oncologist
Impact to fertility, fertility preservation options	“Based on the treatments that we believe are the best for that person, we would then proceed with what does the literature tell us about possible impacts on fertility.”	
Impact to fertility, fertility preservation options	“When I discuss the risks of side effects and the benefits of chemotherapy, I also do discuss the risk of impact on future fertility. My general spiel is that the chemotherapies that we give are to slow down any rapidly dividing cells, like the tumor cells that are in your brain, but there are cells in your body that normally divide rapidly, or that are supposed to have the potential to divide in the future. These include our reproductive cells, or the eggs and sperm that we would eventually use, that would eventually become a baby, and I cannot guarantee that after receiving chemotherapy that those cells will still be healthy enough to have a healthy baby. So, if you are interested in starting a family in the future, we do have resources for fertility preservation. I do discuss with the patient that, for brain tumors, we don’t have a cure yet, for pretty much all of them, and so, we can go through the fertility preservation, but there may be a time when their plans for the future may change, where they won’t be focusing on the family, but there will be some adjustment to the plan, or some recurrence of tumor that may influence their views on things.”	Medical oncologist
Impact to fertility, fertility preservation options, referral to social work/fertility specialist	“As we move forward in our therapy, as we start to initiate our chemotherapy, I want you to keep in mind that some patients will have secondary effects from the chemo on their eggs, or on their sperm, and because of that, I like to warn ahead of time, I cannot guarantee that after this process, your eggs will still be as healthy as they were when we first started. So, if you’re interested, I can get you in touch with one of our fertility preservation experts, or one of our social workers, to discuss what the process might be, in order to plan for that future, if you are interested in having a family. Usually, the process would involve the collection of eggs or sperm before we start treatment, before we expose your body to the chemotherapy, and ideally, we would like to time this appropriately so that there would be no delays in starting your cancer therapy in order to collect your eggs or your sperm, but we can certainly get you in touch with the specialist.”	
Impact to fertility, referral to fertility specialist	“With the initial consultation, the first thing is to educate them on how their treatment may cause infertility. We go over the treatment, which is usually some type of chemotherapy and I always let them know that chemotherapy will have a potential to cause permanent infertility up to a third of the time, 25 to 30-something percent, make sure they know that infertility is possible, and see where they’re at in their life as far as childbearing. If they’re done childbearing, then it’s not that big a deal. But if they’re not even married yet, then I would say it’s a big deal, and so if they are interested in childbearing, we try to get them over to a fertility specialist immediately….I think the important thing in talking about these issues with them is that they understand that no matter what we do, the infertility can be permanent, so it’s important to get to the fertility experts.”	Medical oncologist
Impact to fertility, referral	“I first discuss about the risks of chemo, go through a lot of potential toxicities with chemo. Towards the end of the discussion, I bring up fertility as an issue because I want that to be the last thing we talk about, make sure I make the right referrals if they’re interested. I tell them those same numbers I told you, a quarter to a third of patients can be infertile, the majority of patients are infertile during treatment…Beyond that, if they are interested in having kids I let them know that there’s a quarter to a third percent chance that they may be permanently infertile, even after the chemo is done. Somewhere over the next year after chemo is done, fertility may return but it may not be 100%, and so they have to be ready to be permanently infertile, and if they are interested in childbearing, then at that point, I make the recommendations to go see these other doctors.”	
Fertility-sparing options	“If I talk about chemo and it’s somebody who is otherwise fertile and still interested in childbearing, then it’s one of the first things we get out of the way. If it’s a female, then oftentimes I’ll talk to them about doing other things during chemotherapy. For example, I put them on Lupron just as a hormonal inhibitor to try to shut down the ovaries so that the ovaries don’t get exposed to chemotherapy as much.”	
Impact to fertility, fertility-sparing options, fertility preservation options	“I always let them know what the different treatment options are and what both the potential fertility outcomes are and again, if they don’t have fertility-sparing options, just let them know what their egg freezing options would be, and I always make sure to include discussion of what their preferences are and what they’re currently thinking about their future fertility. Even if they say they are not interested in preserving fertility, the conversation might be much briefer. But we’ll still make sure that they know what kind of an impact [treatment] would have on their fertility, and that they do have options and choices if they wanted to discuss both further.”	Gynecologic oncologist
Impact to fertility	“Mainly anticipated infertility from treatments, their age, perhaps sex, if they have children or would like to have children in early discussions, I think that’s mainly it.”	Medical oncologist
Referral to AYA program	“We’d talk a little bit about your diagnosis and about your prognosis and your treatment plan. It’s important to discuss that some of the treatments that we discussed may impact your fertility. Moving forward, I’m not sure if that’s something that you’ve considered, if you have children, if you’re planning to have children, and then I usually wait for them to go back and tell me, maybe ask some follow-up questions depending on their responses. Then if they suggest that they are interested, I can say we can provide some resources, we have the AYA group that can get in touch with you, and if you are interested in this, we probably should do so prior to your treatment, we would need to do it prior to any treatment so that it wouldn’t impact the samples that may be preserved.”	
Impact to fertility, speed	“For example, a female of childbearing age, I’ll mention one, the potential cryogenic effects of certain chemotherapy, and also if they’re going to be getting radiation impact on fertility, if they’re going to be having surgery of course, like an oophorectomy, where it may completely alter things. I don’t really see as many patients with this, but for patients who have testicular cancer that are going to be having surgery or going to be having chemotherapy, then I think those things can be a little bit challenging, so usually in the hospital and there’s a short timeline.”	
Impact to fertility, fertility preservation options	“On our end, we’ll talk about what we know, at least so far, about fertility, and how chemotherapy may impact fertility, or radiation, I think from a radiation oncology standpoint, and then offer if there is some kind of preservation or banking.”	
Fertility-sparing options, referral	“So it depends, but if it’s a female, we’ll try and discuss cryopreservation, we’ll try and refer over to gynecology. Sometimes I think they’ll consider leuprolide or something like that for ovarian preservation. For men, it would usually be sperm banking.”	

^*^ Quantity does not equate to quality, and longer quotes do not necessarily reflect more comprehensive counseling

One medical oncologist stated that they may prescribe a hormone inhibitor to protect AYAs from the effects of treatment on their fertility, while another reported counseling AYAs on specific impacts to fertility caused by cancer treatment, such as developing menopause for females or azoospermia for males. Another gynecologic oncologist stated:

I always let them know what the different treatment options are and what both the potential fertility outcomes are and again, if they don’t have fertility-sparing options, just let them know what their egg freezing options would be, and I always make sure to include discussion of what their preferences are and what they’re currently thinking about their future fertility. Even if they say they are not interested in preserving fertility, the conversation might be much briefer. But we’ll still make sure that they know what kind of an impact [treatment] would have on their fertility, and that they do have options and choices if they wanted to discuss both further. (Gynecologic oncologist)

Some oncologists mentioned counseling AYAs on the speed with which fertility preservation has to occur (i.e., quickly), that they “would probably wait about two weeks” for an AYA to preserve fertility (Medical oncologist), trying to get AYAs into fertility preservation “as soon as possible” to be able to begin treatment (Medical oncologist), aiming not to delay beginning cancer treatment, and the “short timeline” they are working with (Medical oncologist). Some reported discussing the “risk/benefit ratio” with patients, in which they discuss possible impacts of delaying initiation of cancer treatment to afford time to preserve fertility (Medical oncologist). In addition, some oncologists reported explicitly telling AYAs that fertility preservation needs to be done before treatment and ensuring that they understand that possible infertility after treatment can be permanent.

Few oncologists reported providing AYAs with the following additional information during their fertility discussion: avoiding becoming pregnant during treatment and possible impacts to fetal development should they become pregnant, the effects of treatment on their hormones, and the importance of using contraception during treatment (Supplemental Table 1). One oncologist also reported involving genetic counseling for AYAs with genetic syndromes because this can impact their decision-making regarding fertility.

**Oncologists shared information that they always provide to AYAs during fertility discussions and information they provide on a case-by-case basis **(Table 3). Oncologists’ responses varied when asked what they always include in their fertility discussions. Some reported always discussing future risk of infertility, giving AYAs an option to speak with another provider about fertility, providing AYAs with fertility preservation options and resources (e.g., for sperm banking), and wanting AYAs to understand that fertility preservation is something they can consider without having a significant other. For example, one oncologist shared:

Always mention that there’s a risk of infertility in the future and we obviously can’t risk stratify or give you a percentage necessarily, but they definitely need to be aware and it’s something we need to document as well, and then always obviously give them an option to speak with someone, like social work, about the options there. (Medical oncologist)

**Table 3:** Information always mentioned or mentioned on a case-by-case basis during fertility discussions

	Quote	Oncologist
Always included in fertility discussions	“I always just try to make sure that patients understand that this is something they can consider whether or not they have a significant other, or whether or not they’ve thought about it before. It’s a special circumstance that they’re now being forced to think about it, because of the diagnosis they’re faced with. So I always just encourage them, it may take them by surprise because they hadn’t thought about it before, but it’s certainly something that’s reasonable to consider. So I try to make sure that they understand that they shouldn’t feel ashamed about it, or pressured about feeling one way or another. It’s just something that they should at least hear and can decide how they feel about it one way or the other.”	Medical oncologist
	“Always mention that there’s a risk of infertility in the future and we obviously can’t risk stratify or give you a percentage necessarily, but they definitely need to be aware and it’s something we need to document as well, and then always obviously give them an option to speak with someone, like social work, about the options there.”	Medical oncologist
	“I think if there is a possibility of losing their fertility, I would always mention it. Like say even if we’re doing ovarian cystectomies, I would always mention to the patient that there is a possibility when we take out a cyst from the ovary that they could lose the ovary, benign or malignant, and that’s definitely not usually the case, but I think that if you’re operating near the ovary, you could bleed, you could find something unexpected. Usually the reason you’re operating is because you want to rule out cancer. So if it’s cancer, that ovary is going to come out. So, I think all those things need to be discussed, and depending on what you’re doing, it depends on exactly what you would discuss with them, but I don’t think there’s anything I would never discuss with them.”	Gynecologic oncologist
	“I always include the risks of becoming infertile. Also the risks of conceiving while on therapy. I also introduce the options to preserve fertility and I will offer them a referral to infertility and also I would refer them to AYA if I don’t have the time to discuss all the options.”	Medical oncologist
	“I try to talk about at least have the same general conversation with everyone.”	Medical oncologist
	“The resources, the brochure for the California Cryobank.”	Medical oncologist
	“I generally always try to include what we know based on the literature and even to a certain extent based on my own personal experience as well.”	Medical oncologist
	“I always include that our goal with cancer treatment is to preserve you as you are and to give you more time to do the things that you love and are passionate about. For some, that may involve spending more time with their parents. For some, that might involve being well enough to travel the world, and for others, that involves starting a family of their own. So, our role as the neurooncology team is to help you achieve whatever is a priority for you in the time that we do have. So, we are here to help. Just let us know how we can help you.”	Medical oncologist
	“I always include the potential for permanent infertility. I want to make it clear that they go through treatment, they may not be able to have kids at all ever.”	Medical oncologist
	“I always let them know what the different treatment options are and what both kind of the potential fertility outcomes are on that as well as what cancer treatments are other than that, and again, if they don’t have fertility-sparing options, just let them know what their egg freezing options would be. I always make sure to include, again, that discussion of what their preferences are and what they’re currently thinking about their future fertility. Even if they say they are not interested in preserving fertility the conversation might be much briefer, but we’ll still make sure that they know what kind of an impact it would have on their fertility, and that they do have options and choices if they wanted to discuss both further.”	Gynecologic oncologist
	“I usually mention that everything we discuss is confidential, so that they feel safe in discussing it, that they ultimately can make the decision, that it won’t impact their treatment. I’m not sure what else I always include. I usually give them time and say you don’t have to make the decision today, take some time. If you have follow-up questions, and if I can’t answer them, we can find somebody that will. I discuss the limitations in our understanding about it.”	Medical oncologist
Included in fertility discussions on a case-by-case	“I do discuss prognosis on a case-by-case basis, because I think prognosis will weigh in a lot on an individual’s perspective on what their goals and what their outcomes will be. I mean, we used to think that all brain tumors were alike, and they were all just a death sentence, but now we know that each brain tumor is unique. Even someone who has a brain tumor diagnosis, like glioblastoma could have a different outcome than another patient who has the same glioblastoma. Just like how one patient’s DNA is different from another person’s DNA. So, I think that is a very tailored discussion to have with each individual, not only in terms of the type of tumor and the prognosis that goes with it, but what each person’s goal for the time that they have remaining is.”	Medical oncologist
	“I don’t think there’s really a case-by-case basis. All chemo can have that potential. I usually talk to them in a similar manner. I don’t know that there’s any particular subset of patients that I would treat differently, whether they’re rich or poor, black or white. They all get the same talk.”	Medical oncologist
	“I think egg freezing is definitely a case-by-case basis and really only applies to patients who are either going to get radiation or chemo or need to have both of their ovaries removed, but that doesn’t apply to all of my cancer patients. I mean, things like hormonal therapy as opposed to surgery or radiation or chemo, I discuss it with, on a case-by-case basis, and look at a fertility-sparing option or, particularly endometrial cancer patients. And then really for cervical cancer patients, there’s just [INDISTINCT] surgical options if they want fertility or not, and we need to discuss those, kind of before…I do think that the discussion is a little different if patients already have multiple children than if they haven’t had any kids, and potentially kind of where they are in the age range and kind of relative to how bad their cancer is and how much a fertility-sparing option makes sense in their overall treatment plan, but yeah, I would still discuss it. I think just how I would recommend it or what my counseling would be might vary a little bit.”	Gynecologic oncologist

Oncologists reported discussing patient prognosis, fertility-sparing options, and fertility preservation with AYAs on a case-by-case basis. Some mentioned that they have the same general conversation with everyone and that a case-by-case basis for fertility discussion does not exist because all chemotherapy has potential for infertility. For example, one gynecologic oncologist stated:

I think egg freezing is definitely a case-by-case basis and really only applies to patients who are either going to get radiation or chemo or need to have both of their ovaries removed, but that doesn’t apply to all of my cancer patients. I mean, things like hormonal therapy as opposed to surgery or radiation or chemo, I discuss it with, on a case-by-case basis, and look at a fertility-sparing option or, particularly endometrial cancer patients. And then really for cervical cancer patients, there’s just [INDISTINCT] surgical options if they want fertility or not, and we need to discuss those, kind of before…I do think that the discussion is a little different if patients already have multiple children than if they haven’t had any kids, and potentially kind of where they are in the age range and kind of relative to how bad their cancer is and how much a fertility-sparing option makes sense in their overall treatment plan, but yeah, I would still discuss it. I think just how I would recommend it or what my counseling would be might vary a little bit. (Gynecologic oncologist)

**Some oncologists reported discussing cost with their AYA patients** (Supplemental Table 2). One medical oncologist described the costs of fertility preservation as “enormous” and shared that they are “giving the warning that that has to be part of this consideration” (Medical oncologist) while another reported discussing cost specifically with AYAs who have limited resources. When asked how they determine which patients have limited resources, they explained:

Well, I usually ask, and most of the time, in patients that have limited resources, they will mention how much does it cost and it will be the first question that comes out of their mouth and then I kind of understand that that’s something that gives them a lot of anxiety. (Medical oncologist)

Regarding cost and insurance, once gynecologic oncologist emphasized:

If one day insurance would pay for this, that would be super ideal because it’s not like they chose to have cancer and they chose to be losing their fertility. It’s part of their healthcare. (Gynecologic oncologist)

**Though responses varied, oncologists shared information they avoid mentioning or feel is not necessary when discussing fertility **(Table 4). One gynecologic oncologist shared that AYAs don’t need to tell them they aren’t interested in future fertility because they would discuss with them anyway. A medical oncologist reported that going into technical details regarding the fertility process was not necessary because they [the oncologist] provide patients with general information but “don’t really know what the fertility doctor does” (Medical oncologist).

**Table 4:** Information oncologists avoid mentioning or feel is not necessary when discussing fertility

	Quote	Oncologist
Not necessary	“I guess judgment. We always have to suspend judgment. Every person has their own life plans. Cancer throws a wrench in things, so you have to suspend judgment, even if you have a patient who’s 45 and wants to preserve fertility, it’s a conversation that as a clinician, you should have. You owe it to them to have, and so I try to teach this to my fellows and say yes, it’s less realistic that they’re likely to be able to either freeze an embryo or even to maintain their fertility beyond that, given their age. It’s still a conversation that they have every right to have.”	Medical oncologist
	“I’m trying to think of the conversations I’ve had. Nothing that was unnecessary that I can think of.”	Medical oncologist
	“I think most of its necessary. I don’t know if I can think about anything that’s not necessary. I mean, if a patient has questions about it, and if you’re talking about, like I said, removal of any of their reproductive organs, I think anything’s fair game and there’s nothing that’s off the table or that I would avoid discussing.”	Gynecologic oncologist
	“I think the most important thing is that you get the information out there, you tell them what the risk factors are, you tell them what the quotable percentages are, that they can become infertile. Like I said, I don’t have anything that’s off-limits for discussion. I can’t think of anything that’s off limits.”	Medical oncologist
	“I wish that finances were not necessary [when discussing fertility], or insurance were not necessary, but it’s a reality that we just have to deal with, I think. Like that patient that I mentioned, my recent patient whose cancer treatments were delayed because of insurance authorization delays. I wish that that did not happen, but I think that, unfortunately, it’s built into the system, that we have to jump through all these hurdles to get patients the care, the preventative care, the current care that they need.”	Medical oncologist
	“For me, I don’t think it’s necessary for me to go into the details of the actual process. I give them some general ideas but to be honest I don’t really know what the fertility doctor does. I know at least for women there’s a lot of high doses of hormones to stimulate the ovaries. So I might give some ideas about that. Sperm banking for men is a little bit more straightforward, but I don’t know that I really get into too many details on the technical aspect of it.”	Medical oncologist
	“It is not necessary for a patient to tell me straight out that they want future fertility. Like I think if they say no, they don’t want kids, I still kind of discuss it anyways. In the AYA population, I think mostly because I just want to make sure that that discussion is had before they make decisions that will impact their fertility. I just want to make sure that they know and understand all of the implications of the choices they’re making, and I always kind of ask where they’re at with like, ‘Do you have kids? Do you want more kids? Have you thought about it,’ like if they have a timeline, but kind of regardless of the answer, I’ll still talk about it. I just kind of bring the discussion around what they’re currently thinking at that point in their life.”	Gynecologic oncologist
	“I guess imposing our opinion on it is not necessary, maybe trying to remain objective.”	Medical oncologist
Avoid mentioning	“There’s the occasional case where a patient will sort of ignore your advice and they will have a pregnancy on therapy, and normally…well, every time that’s happened, which has been a handful of times, and believe it or not, the baby came out normal. So, thank goodness, right? But at the same time you don’t want to encourage that behavior. So I will not mention that there have been occasions where they’ve actually… Unless of course they actually conceived, and then they’re distraught, and I can say that, well, if they’re determined to carry this to term, I can tell you that some of them have not, but I would get with your gynecologist or your obstetrician to counsel them on that.”	Medical oncologist
	“I guess try not to focus on the cost because there’s some financial assistance and some resources. I think the Lance Armstrong Foundation and such and the social workers help with that also.”	Medical oncologist
	“I mean I think just by the nature of the discussion, it adds another layer of stress of course to the patient. I don’t think there’s anything that we would necessarily leave out.”	Medical oncologist
	“Nothing. I’m a cancer specialist, so the whole discussion can be upsetting, but there is nothing I avoid talking about.”	Medical oncologist
	“I generally when I interact with patients, I try to be completely transparent. I’m not a person who will hide anything. So there’s probably not too much I’m going to avoid unless it’s something that’s not really relevant or something like that. I can’t think of anything.”	Medical oncologist
	“As much as I discuss prognosis with them, I avoid bringing up specific concrete numbers. For instance, I will only say that a patient with glioblastoma, their median survival would be under two years. I avoid saying that under two years phrase, unless they specifically ask me how much time do I have. Or, when I say it, I’ll phrase it as, these are all the statistics compiled from hundreds of thousands of patients, but in the end, these are just numbers, and you are more than a number, you’re more than a statistic. So, I try to encourage them that yes, we do have data, and I need to make you aware of that data, but I don’t want you to live by that data, and just be counting down that I have two years minus one day, two years minus two days, as they’re living their lives.”	Medical oncologist
	“I don’t think there’s anything I truly avoid…I guess the one thing I do bring up but I don’t stress is prognosis.”	Medical oncologist
	“I avoid making it like, ‘This is what you have to do, and you’re just going to, you know, lose your fertility. And that’s it,’ you know? Like I much more try to give options and choices and like involve them in the process. I think I’ve had patients who had previously seen somebody before they came to me, for example, and were very upset about being told that they were just, ‘You need a hysterectomy,’ and that was it. Or they just needed their ovaries out, that was it, and didn’t have a discussion about fertility-sparing options. So, yeah, I think my approach is different, and I’m just trying not to be so harsh about it, or so black and white about it, even if I think that losing their fertility is just something that has to happen as part of their best cancer treatment.”	Gynecologic oncologist
	“I’m not sure. I’m usually pretty frank [LAUGHS] because I think it’s important for patients to know.”	Medical oncologist

Several oncologists added that they have “frank” discussions with patients, and that there is nothing they would never discuss or avoid. One oncologist reported not focusing on cost during fertility discussions because some financial assistance options are available for patients. Oncologists also reported avoiding providing patients with concrete numbers regarding prognosis, avoiding encouraging conceiving while receiving treatment, and avoiding making patients feel like they don’t have autonomy in their decision-making.

## Discussion

While there is consensus that fertility discussions are the standard of care for cancer patients with reproductive potential [5–7, 9–11, 31], their implementation in practice has seldom been thoroughly explored. This study provides a detailed description of information delivered by oncologists to their AYA patients during fertility discussions. Oncologists described a variety of information they deliver to AYAs during fertility discussions, suggesting the lack of a standardized counseling framework. Overall, findings begin to unveil the nuances surrounding fertility discussions and can contribute to intervention development to improve the provision of guideline-concordant oncofertility care and enhance patient-provider communication for this at-risk population.

Despite ASCO clinical practice guidelines detailing necessary information and serving as a benchmark to inform fertility discussions (i.e., infertility risk, referral to a reproductive specialist, preservation options, costs, pregnancy risks) [5–7], the variable delivery of information suggests that fertility discussion content is unstandardized. While exploring counseling specific to patient cancer type and/or oncologic specialty was beyond the scope of this study which sought to detail counseling, broadly, for the AYA patient population, our findings on variability in discussions suggest that counseling may vary depending on provider specialty. As we did not seek to derive conclusions based on patient or provider characteristics, we encourage future research to explore oncofertility counseling within silos of cancer types, treatment patterns, and/or oncologic specialties to gain a deeper understanding of variability in counseling. Ultimately, our findings suggest that oncofertility counseling that incorporates all elements of guidelines appears infrequently delivered, and strategies to standardize fertility discussions to include recommended information are needed for AYAs to receive actionable counseling that informs decision-making regarding their reproductive futures.

Only some oncologists in this study reported counseling AYAs on costs associated with fertility preservation, despite financial barriers frequently reported as cause for the underutilization of such services and ASCO guidelines detailing the need for such counseling [5–7, 23, 32–34]. AYAs report feeling unprepared and frustrated due to a lack of financial-related communication, experience more financial hardship, are likely to have competing financial concerns (e.g., student debt), and are more often un- or under-insured than older patients, making fertility preservation highly prohibitive [35–39]. High costs, particularly for females, are further compounded by a lack of insurance coverage [32, 40–44]. Fortunately, substantial legislative advancements surrounding insurance coverage for fertility preservation have passed in recent years, with 22 states enacting laws regarding insurance coverage for fertility across the USA (with variability in state mandates for insurance coverage including in vitro fertilization, fertility preservation, and/or infertility as of September 2024) [45, 46]. Legislative improvements will continue to mitigate the cost-prohibitive nature of preservation, making it more accessible to patients. As financial discussions between AYAs and providers are associated with decreased financial toxicity [35], comprehensive discussions including cost that guide AYAs in leveraging available resources (e.g., maximizing insurance coverage) will become even more essential to increasing access to fertility preservation for this population.

Barriers to fertility discussion are well-documented and may be related to the variability in discussions reported by oncologists in the present study. Provider-level barriers include knowledge, comfort, self-efficacy, biases or perceptions regarding fertility preservation (e.g., that it is secondary to cancer treatment, the difficult nature of the conversation, outcome expectancy), and concerns regarding the high costs of preservation [47–49]. As AYAs report fertility specialists or oncologists as their preferred source of information on cancer-related late effects, mitigating barriers to discussion, particularly on the provider level, and establishing clear referral pathways to fertility specialists such as reproductive endocrinologists are imperative to facilitate quality care delivery [50]. Several training programs, including Enriching Communication skills for Health professionals in Oncofertility (ECHO) and Educating Nurses about Reproductive Issues in Cancer Healthcare (ENRICH), have been developed to facilitate the delivery of comprehensive oncofertility counseling [51, 52]. As the field of oncofertility continues to develop and fertility preservation becomes more accessible to patients, alleviating barriers to arming patients with actionable oncofertility-related knowledge will become more important than ever.

This study has strengths and limitations. The non-random recruitment of oncologists who practice at a single NCI-designated comprehensive cancer center in California with state mandated insurance coverage for fertility services (which did not apply to Medi-Cal managed plans at time of data collection) reduces generalizability to other cancer care delivery contexts [46, 53]. While oncologists were required to report treating AYAs for study eligibility, AYAs did not comprise the primary population treated for any participating oncologist and their knowledge or care delivery may be impacted by limited exposure to patients of reproductive age. Despite these limitations, these findings provide the first open-ended qualitative exploration of fertility discussion content and begin to explore adherence to clinical guidelines in practice. Furthermore, findings are reflective of care at an NCI-designated comprehensive cancer center, representing gold standard care in the USA, identifying areas in which highly resourced care systems may be lacking in care delivery [54]. Additional complementary quantitative research closely mapping fertility discussions to clinical practice guidelines is needed to determine their level of guideline-concordance. Further, the extension of this research to less resourced environments is essential. In aggregate, such research may inform how actionable oncofertility counseling is for AYAs across diverse care settings and may reveal additional patient- and provider-reported barriers to the provision of reproductive health guidance. Further, additional research pairing patient and provider perspectives using a dyadic approach may yield valuable results with respect to patients’ perspectives and preferences regarding oncofertility counseling received.

### Conclusion

This study qualitatively explores fertility discussions between oncologists and their AYA cancer patients from the perspective of oncologists by comprehensively describing oncofertility counseling at an NCI-designated cancer center and identifying patterns and gaps in care delivery. As the primary source of healthcare information [55], it is critical that oncologists delivering gonadotoxic cancer therapies adequately inform AYAs of possible treatment-related infertility. However, fertility discussions appear unstandardized, with variability in their depth and content, suggesting limited comprehensive adherence to oncofertility clinical practice guidelines. Such heterogeneity likely translates to varied patient understanding of their fertility status, methods to seek fertility preservation, and an increase in reproductive concerns. Interventions including provider training to increase provision of comprehensive, guideline-concordant counseling are needed to provide actionable pathways by which AYAs can proactively mitigate adverse reproductive health outcomes.

## Electronic supplementary material

Below is the link to the electronic supplementary material.

Supplementary file1 (DOCX 21.4 KB)

## Data Availability

The data that supports the findings of this study are available both in text and in the supplementary material of this article.

## Appendix Semi-structured interview guide

I. Fertility discussions
  1. Tell me about the background of the patients you typically see. *(Demographics)*
    a Approximately what percent of patients do you see that are diagnosed with cancer as adolescent and young adults (AYAs) under 40 years?
  2. At what points in the continuum of care does fertility come up with AYA patients?
    a How far in advance before treatment begins?
  3. Walk me through what care looks like when an AYA’s treatment may impact their fertility.
    a Who is typically initiating these conversations? *(Counseling, referral)*
    b Who on your team talks to patients about their fertility? (*Person responsible*)
    c Who on your team has training or experience in fertility or reproductive health?
  4. How do you personally feel about the process of discussing fertility with patients?
  5. What prevents you from discussing fertility with AYAs or makes fertility discussions difficult?
    a What makes you hesitant to discuss fertility with AYAs? *(Barriers)*
  6. Who supports you in caring for AYAs who are likely to have treatment-related infertility?
    a Do you need more support? If so, from who?
II. Knowledge and implementation of guidelines
  - 7. Knowledge and implementation of guidelines
    a Do you discuss fertility with every AYA?
    b Do you discuss based on personal perception of patient risk?
  - 8. What informs your discussions of fertility with AYAs? (*Guidelines, institution, patient needs)*
  - 9. How do you stay up to date on discussing fertility with AYAs? *(Outside resources, seminars)*
    a How do you learn more about discussing fertility?
    b What would you like to know more of?
    c What do you think is not necessary when discussing fertility?
  - 10. Imagine I am an AYA and am likely to have treatment-related infertility. Walk me through how you start a fertility discussion, what you say throughout, and how you conclude this discussion.
    a What do you always include in your fertility discussions with patients?
    b What do you discuss on a case-by-case basis, dependent on patient circumstances?
    c What do you avoid mentioning to prevent upsetting patients?
  - 11. What external clinical practice guidelines exist for fertility discussions? *(Not institutional)*
    a Who creates these guidelines?
    b Tell me about why these guidelines may or may not be necessary.
  - 12. What is your perception of existing guidelines?
    a How do you integrate existing guidelines in discussions of fertility with AYA patients?
    b What would make you more likely to include components of guidelines in your discussions?
  - 13. In what way do existing guidelines inform your fertility discussions?
    a How do they help fertility discussions with AYA patients? *(Solutions, what works)*
    b How do they hinder fertility discussions with AYA patients?
    c What changes would you like to see surrounding fertility discussions or the systems in place?
  - 14. What are the policies or protocols in place at your practice to support fertility discussions in line with these guidelines?
    a Institutional regulations (How do you work around not having regulations?)
  - 15. What helps you have discussions that include the components of guidelines?
    a What do you need to implement these guidelines in your discussions?
  - 16. What would make you more likely to refer patients to fertility specialists on a regular basis?
