# Supplementary material for: Behind closed doors: a qualitative study exploring the content of fertility discussions between oncologists and their adolescent and young adult cancer patients from the perspective of oncologists at an NCI-designated comprehensive cancer center
Source: Support Care Cancer. 2025 Mar 21;33(4):308. doi: 10.1007/s00520-025-09269-0 (PMC11925993; doi:10.1007/s00520-025-09269-0)
Supplement: Supplementary file 1 — Supplementary file1 (DOCX 21.4 KB) [file 520_2025_9269_MOESM1_ESM.docx]

| **Supplemental Table 1:** Additional information included in fertility counseling | | |
| --- | --- | --- |
| **Information** | **Quote** | **Oncologist** |
| **Avoiding pregnancy during treatment and possible impacts to fetal development if they become pregnant** | "We wouldn't want them to be trying to get pregnant or getting pregnant during that because that actually could have negative effects on early stages of fetal development." | Medical oncologist |
|  | "Whether it's that they should not try to get pregnant during the time at which we're going to be treating them." | Medical oncologist |
|  | "It's also important that while you're on the systemic therapy that you not try to get pregnant because there can be some abnormalities with the fetus should you conceive, or with the sperm or with the egg, and so, therefore we recommend that you not participate in pregnancy or conceive while you're on therapy." | Medical oncologist |
|  | "The importance of not conceiving during therapy and also really the lack of long-term physical maladies associated with infertility. So, just because you're infertile doesn't mean that is going to affect your heart, your lungs, your brain, or things like that, unless you're menopausal...So I always give them an option and tell them that it doesn't really have a large effect on the rest of your physiological being and getting rid of this cancer is the objective here." | Medical oncologist |
|  | "You have to let them know that if they do become pregnant during treatment that there’s certainly the potential for fetal harm, fetal malformations, fetal demise even, and then we stop chemo. I mean it hasn’t happened to me, but I would stop chemo and let them know that if they decide to carry the child through that they have to be off chemo at least through the first trimester. We don’t have a lot of studies on chemo and pregnancy, but after the first trimester, we believe chemo is safer and we give chemo in the second and third trimester of pregnancy. So they would be able to be treated for a little while anyhow." | Medical oncologist |
| **Hormonal status** | "There is also more to think about in addition to fertility. You have to think about their hormonal status. So that becomes part of the conversation as well, because fertility is one part of it but we're also talking about potentially removing ovaries which make hormones and how that can affect their future and then how we would plan on treating those things." | Gynecologic oncologist |
|  | "We have interventions for chemo-induced menopause, so you can always go on hormone treatment." | Medical oncologist |
|  | "They’ll be on Lupron throughout the course of treatment when I’m treating them." | Medical oncologist |
| **Importance of contraception during treatment** | "We would say this is the time you need to use contraception to make sure that that doesn't come into play as part of this therapeutic journey that we're about to embark on. So it's not that it happens biologically immediately, but it becomes an issue immediately." | Medical oncologist |
|  | "I also tell them that don’t assume that chemotherapy makes you infertile completely. You still need to practice safe sex and barrier protection." | Medical oncologist |
| **Genetic counseling** | "Of course, if they have genetic syndromes, then we make sure to have genetic counseling involved because that can also impact their fertility or planned fertility or making those decisions." | Medical oncologist |

| **Supplemental Table 2:** Counseling surrounding oncofertility costs | |
| --- | --- |
| **Quote** | **Oncologist** |
| "Going as far as egg harvest, or embryo preservation, or anything like that, that's going to all be out of pocket, and the costs are going to be enormous. So I don't discourage them, but I say I'm just giving the warning that that has to be part of this consideration." | Medical oncologist |
| "Especially if you're talking about things like surrogacy or even egg retrieval and IVF, cost needs to be a part of that discussion. Usually if they're even thinking about that I send them to REI for counseling, because the actual cost of these things is going to vary. I usually quote them about $15,000 for IVF. I actually don't know what the price for a surrogate is, but when we start talking about all of those specifics I would rather have them speaking with the reproductive endocrinology specialist at that point. So if they're even thinking about it, I send them there, and sometimes they'll just email the REI doctor if there's any further questions that I couldn't answer myself about cost and things like that. I know that there are some support programs for our patients who have cancer to help them preserve fertility and that will pay for part of the cost, and honestly, if one day insurance would pay for this, that would be super ideal because it's not like they chose to have cancer and they chose to be losing their fertility. It's part of their healthcare.” | Gynecologic oncologist |
| "I talk to them about their chances, how their treatment would decrease their chances, and then tell them about fertility preservation and what that entails, but like financially as well. I think that’s important." | Medical oncologist |
| "If they even just want a consult to get more information, I do tend to discuss the general cost of that." | Gynecologic oncologist |
| "I guess patients who may have limited resources, I do make sure to mention that there’s an associated cost with it because I think that gives patients a lot of anxiety." | Medical oncologist |
| “Well, I usually ask, and most of the time, in patients that have limited resources, they will mention how much does it cost and it will be the first question that comes out of their mouth and then I kind of understand that that’s something that gives them a lot of anxiety. I’ll also let AYA know about that too because I think that there are some groups that can help provide funding for this, or at least they can give some better estimates than I do for how much things cost." | Medical oncologist |
